# Population admixture in Chinese and European *Sus scrofa*

**DOI:** 10.1038/s41598-017-13127-3

**Published:** 2017-10-13

**Authors:** Minhui Chen, Guosheng Su, Jinluan Fu, Qin Zhang, Aiguo Wang, Mogens Sandø Lund, Bernt Guldbrandtsen

**Affiliations:** 1 Center for Quantitative Genetics and Genomics, Department of Molecular Biology and Genetics, Aarhus University, Tjele, Denmark; 2 Department of Animal Genetics, Breeding and Reproduction, China Agricultural University, Beijing, China

## Abstract

Relationships between different populations were investigated using Porcine 60 K data from 1,135 domestic pigs and wild boars across Europe and China. The results indicate that most European breeds have been introgressed with Chinese ancestry, but the extent of introgression varies considerably among breeds. Moreover, the main source of this introgression is pigs from South China, closely related to Bamaxiang and Dongshan pigs. Contributions from East and Central Chinese pig breeds are also detectable. Phylogeny reconstruction places European wild boars among European domestic breeds. Coalescent simulations indicate that this may be the result of gene flow from European wild boars to European domestic pigs. These results will facilitate further genomic studies such as genome-wide association studies, selection signature detection and genomic prediction.

## Introduction

The independent domestication of pigs in western Eurasia and East Asia^1–3^, roughly 10,000 years ago, led to two phenotypically and genetically distinct populations in Europe and China. Subsequent artificial and natural selection has resulted in a wide variety of breeds diverse in appearance, local adaptation and productivity^4^.

In the process of breed formation, the population structure of pigs increased in complexity by gene flows between domestic pigs and wild boars^5^, and hybridization between pig breeds, especially the introduction of Chinese pigs into European commercial breeds. During the Industrial Revolution, Northern European farmers introduced Chinese pigs to improve local breeds to meet the demand of intensified agriculture^4^. Given its implication for the origin of modern commercial breeds, the introgression from Chinese pigs to European breeds has been a subject of great attention. The hybrid origin of European commercial breeds was first demonstrated by Giuffre *et al*.^3^, and subsequent studies of mitochondrial DNA (mtDNA), microsatellite and genome-wide SNP data further confirmed a large proportion of Chinese ancestry in most European breeds^6–8^.

Extensive gene flow between ancestral breeds has obscured the origin of modern breeds and the relationships between them. To date, several investigations on the demographic history and population structure of pigs^9–11^ using mtDNA and genome-wide SNP data have been reported. However, given its extra-nuclear and maternal inheritance, mtDNA may have a demographic history distinct from the rest of the genome. In addition, it is not able to reflect fine population structures or male-mediated gene flows. Another important drawback of mtDNA is that it behaves as a single locus. Consequently, it may not reflect the true relationships between breeds after ~200 years of genetic drift and selection since the major introgression from Chinese pigs to European breeds. Genome-wide SNP data studies provide a feasible approach to getting a more detailed picture. However, previous studies are generally concerned with only a set of representative breeds or breeds within specific geographic regions^8,12^. Therefore, a genome-wide investigation with a wider representation of domestic pigs and wild boars is necessary to detect the divergence and admixture of these populations.

Currently, SNP array genotyping technology is a powerful tool in population genetic studies of animal species^13–15^. With the objective of obtaining a comprehensive characterization of the genetic structure of Eurasian *Sus scrofa*, we carried out a study utilizing the genome-wide SNP data from 153 wild boars from Europe and China, and 982 domestic pigs from 18 European breeds and 13 Chinese breeds. The aim of this study was to explore the divergence and admixture of pig breeds mainly by characterizing the source breeds of the introgression from Chinese pigs into European breeds and quantifying their contributions.

## Results

### Data refinement

We collected the Porcine 60 K data from 1,135 domestic pigs and wild boars, as summarized in Table S1. The dataset comprised of 734 domestic pigs from 18 European breeds, 248 domestic pigs from 13 Chinese breeds, and 153 wild boars from Europe and China. To refine the dataset, we removed samples with closely related individuals.

**Figure 1:**
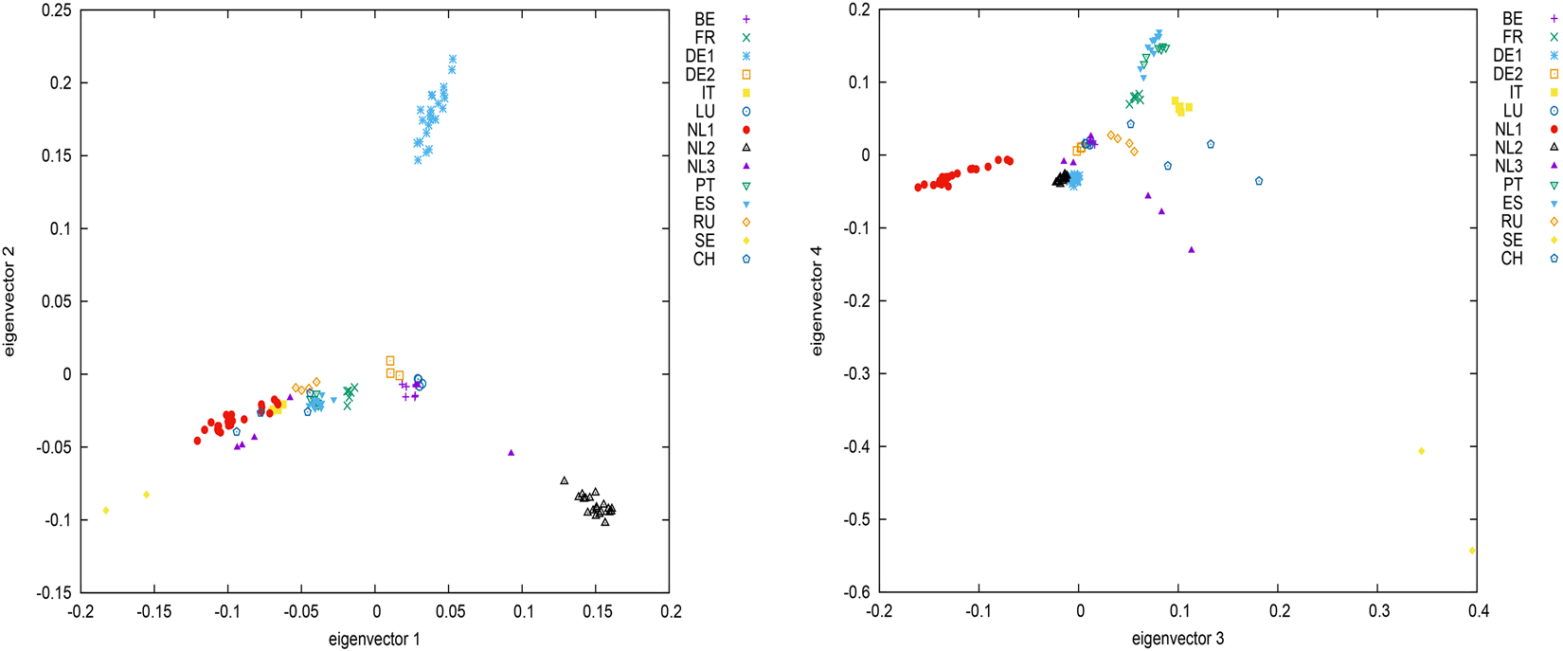
The plot of principal component analysis on European wild boars. The first four principal components explained 6.9%, 5.7%, 5.3% and 4.2% of genetic variance. German wild boars split into two groups, DE1 and DE2. Dutch wild boars split into three groups, NL1, NL2 and NL3. BE: Belgium, FR: France, DE: Germany, IT: Italy, LU: Luxemburg, NL: Netherlands, PT: Portugal, RU: Russia, ES: Spain, SE: Sweden, CH: Switzerland.

Principal component analysis (PCA) on European wild boars (EUW) showed that individuals from each country clustered together as one group, except for wild boars from Germany and Netherlands (Fig. 1). German wild boars separated into two groups; Dutch wild boars clustered into two groups, and the remaining individuals were classified as a third group. According to this result, relatedness tests and subsequent analyses were performed for each European wild boar group separately. After excluding 440 closely related individuals, the dataset remained 695 individuals, including 15 Chinese wild boars, 109 EUW, 430 European domestic pigs (EUD), and 141 Chinese domestic pigs. European pigs included 18 breeds with sample sizes ranging from 3 for White Duroc to 58 for Large White; Chinese pigs included 13 breeds with samples sizes ranging from 4 for Ganxi and Shaziling to 19 for Tibetan pigs. Table S1 shows the change of sample sizes in the process of data quality control and relatedness test.

### ADMIXTURE analyses of Eurasian *Sus scrofa*

**Figure 2:**
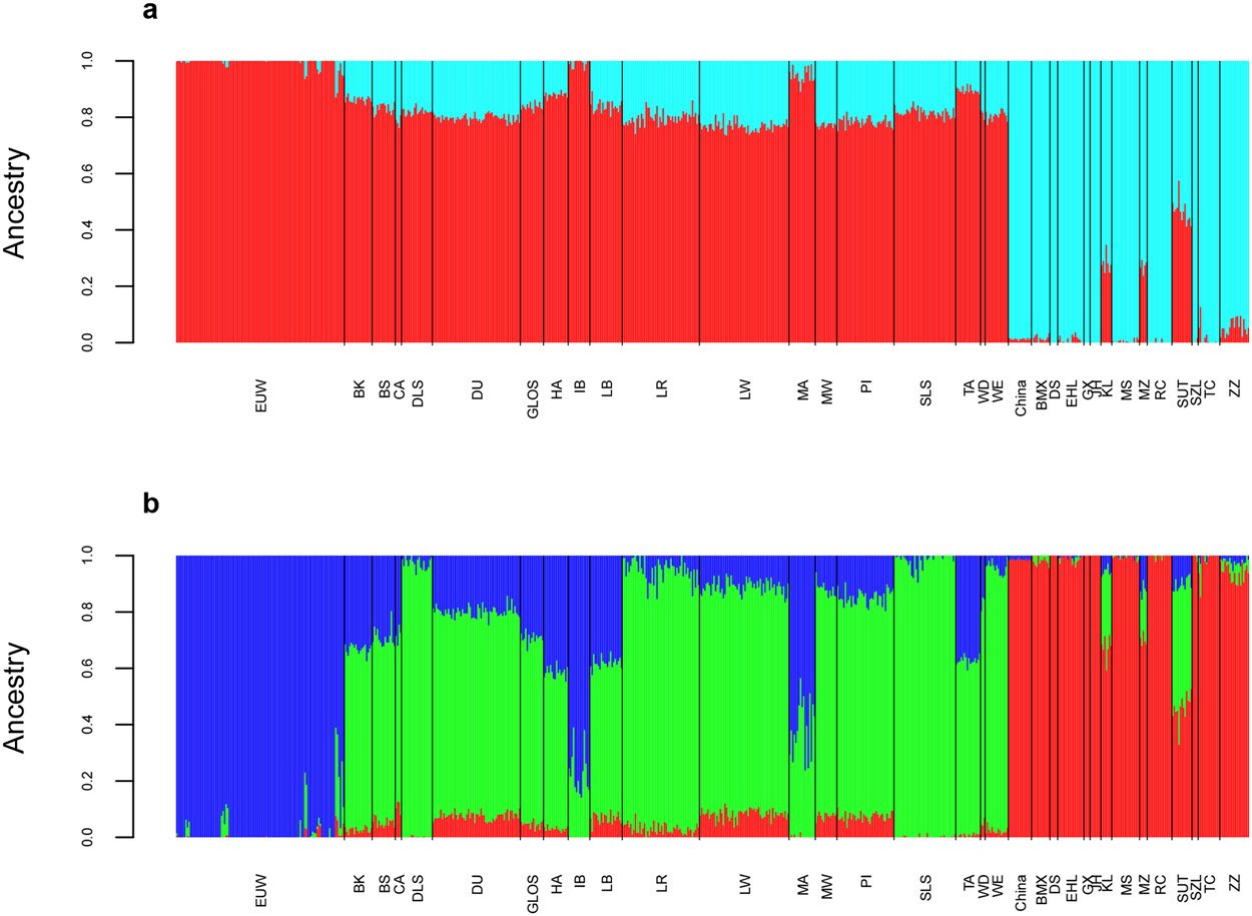
Ancestry composition using two and three ancestral populations (*K* = 2 and 3) in ADMIXTURE analyses.

To investigate the population structure of wild boars and domestic pigs from Europe and China, we first conducted analyses using ADMIXTURE 1.23^16^. The cross-validation error was minimized at *K* = 32 (Supplementary Figs S1 and S2). It indicated a high number of contributing populations, resulted from breed formation, geographic dispersal and artificial selection. At *K* = 2 (Fig. 2), the two modelled ancestries clearly distinguished the ancestors of European and Chinese *Sus scrofa*; at *K* = 3 (Fig. 2), the model further distinguished the ancestors of EUW and EUD. These two models could clearly illustrate the big events of gene flow between European and Chinese *Sus scrofa*. At *K* = 2, the fraction of Chinese ancestry was about 20% in most European breeds, except for two traditional breeds, Ibérico and Mangalica pigs. Therefore, in the three-population test and *F*_4_-ratio estimation, we used Ibérico pigs as a European breed without introgression from Chinese pigs. Moreover, there existed a low level of Chinese ancestry in EUW from Netherlands, Switzerland, Sweden, and Russia. After increasing *K* to 3 (Fig. 2), these EUW also presented a high proportion of the ancestry of EUD. Therefore, we inferred that the gene flow from EUD to EUW led to an indirect introgression from Chinese pigs to EUW. When *K* = 2, three Chinese breeds (Kele, Min and Sutai) showed high percentages of European ancestry. This result confirmed the gene flow from Western pigs into Kele and Min pigs, and the hybrid nature of Sutai pigs between Chinese Taihu pigs and Duroc pigs from a previous study^17^. In addition to these three breeds, Tibetan pigs were also admixed with European pigs. To confirm the admixture of these four breeds, we further conducted the three-population tests for each Chinese breed. We found that only these four admixed breeds identified in the ADMIXTURE analysis had significantly negative *f*_3_ statistics (Table S2).

### TreeMix analyses of Eurasian *Sus scrofa*

**Figure 3:**
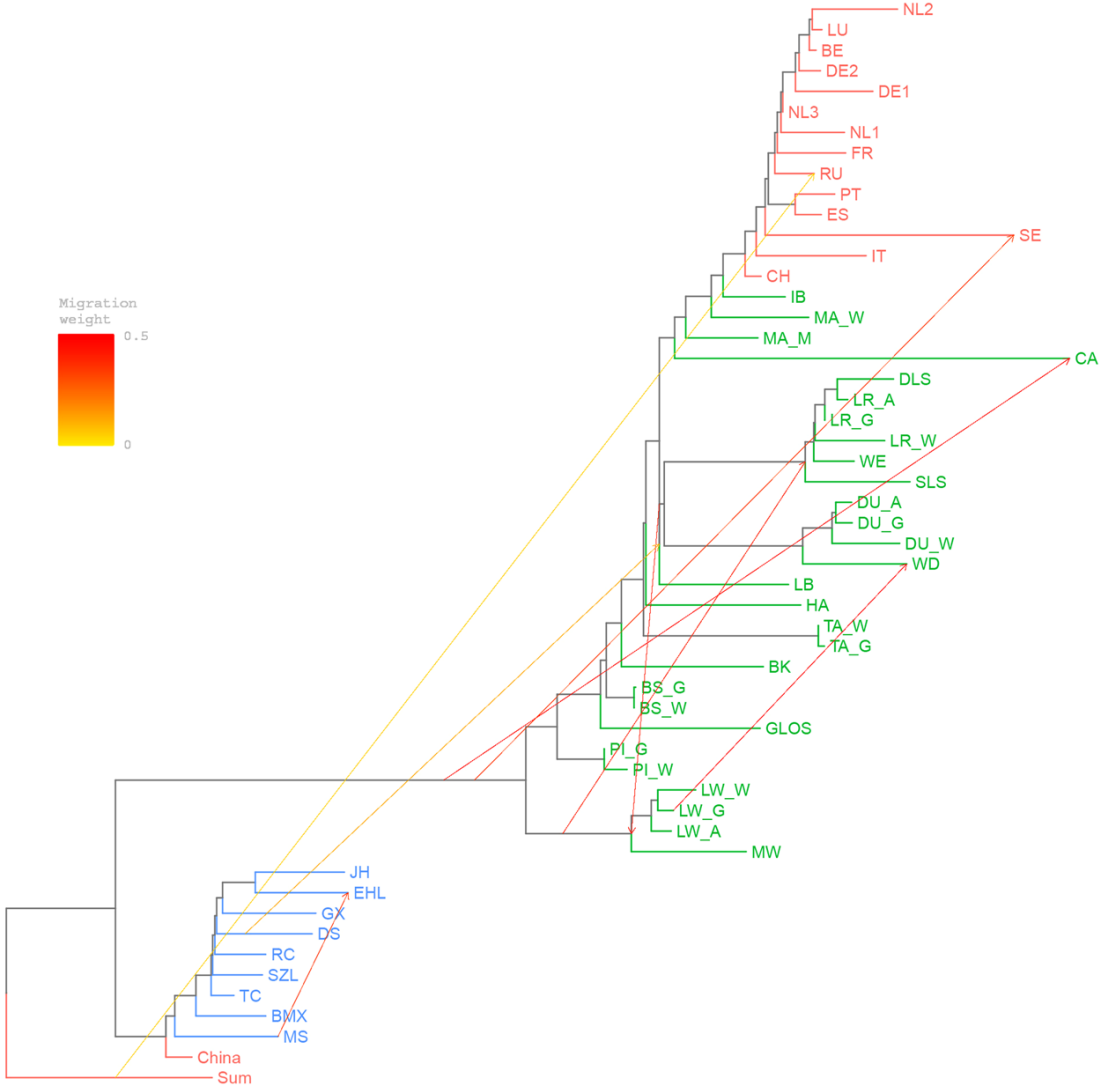
Phylogenetic network of the inferred relationships between populations at m = 8 (eight migration events). Wild boar populations were coloured as red; Chinese domestic breeds were coloured as blue; and European domestic breeds were coloured as green. Migration edges were coloured according to percent of ancestry received from the donor population. Swine breeds with at least two data resources were separated into groups corresponding to the resources. European wild boars were separated into groups according to the result of principal component analysis. Sum represented Sumatran wild boars. BE: Belgium; CH: Switzerland; (DE1, DE2): Germany; ES: Spain; FR: France; IT: Italy; LU: Luxemburg; (NL1, NL2 and NL3): Netherlands; PT: Portugal; RU: Russia; SE: Sweden.

We used TreeMix^18^ to infer the patterns of population splits and admixture in Eurasian *Sus scrofa*, and in particular the introgression from Chinese pigs to European breeds. Supplementary Fig. S3 shows the proportion of variance in relatedness between populations explained by each model. The tree model without migration events explained 98.21% of the variance. At m = 8, the model explained a slightly higher percentage (99.43%) of the variance, and adding more migration events did not account for much more variance. Besides, the model (m = 8) generated acceptable residuals between populations (Supplementary Fig. S4). The split between Chinese and European *Sus scrofa* was consistent in all trees, and pig groups of the same breed always clustered together. At m = 8, we found one significant gene flow from Chinese Dongshan pigs to European breeds, including Large Black, Duroc, White Duroc and Landrace (Fig. 3). In tree models with 2 to 10 migration events (Supplementary Fig. S5), we all observed the gene flow from Dongshan to EUD, except for m = 6. To evaluate the consistency of migration edges, we repeated the analysis with eight migration events 10 times with different random seeds (Supplementary Fig. S6). All independent runs of TreeMix inferred migration from Chinese pigs to Duroc, Landrace, Welsh, Large Black, Danish Landrace and Pied Landrace. Besides, we also identified two admixture events that were consistent across all independent TreeMix runs: an introgression from Sumatran to Russian wild boars and an introgression from the common ancestor of European *Sus scrofa* to Swedish wild boars.

To confirm the introgression from Chinese pigs to European breeds, we calculated the *f*_3_ statistics for each European breed. Table S2 shows all breeds with significantly negative *f*_3_ statistics. There were significant evidences of gene flows from Chinese pigs to Large White, British Saddleback, Landrace and White Duroc. This result matched well with the tree allowing eight migration events in the TreeMix analysis, which inferred genetic contributions from Chinese pigs to European breeds, including Landrace, White Duroc and Large White, across several replicates (Fig. 3 and Supplementary Fig. S6). We also observed that for all of these four European breeds with significantly negative *f*_3_ statistics, the most significantly negative *f*_3_ statistics were obtained when Bamaxiang or Dongshan was used as one source population. It was consistent with the results of TreeMix analyses (m = 8) where Dongshan and Bamaxiang were the main source of the introgression from Chinese pigs to European breeds. As both Dongshan and Bamaxiang come from Guangxi Province in China, we treated Bamaxiang, which had bigger sample size (n = 12), as the representative contributor of the introgression from Chinese pigs to European breeds. We used Bamaxiang in *F*_4_-ratio estimation to calculate the mixture proportions of the four admixed European breeds (Supplementary Fig. S7). The median proportions of Chinese ancestry were 28.7%, 27.0%, 24.5% and 21.8% in Large White, Landrace, White Duroc and British Saddleback.

To check the consistency of ancestry composition across chromosomes, we conducted the three-population tests for each European breed using the five chromosomes with most markers (Supplementary Fig. S8), including SSC (*Sus scrofa* chromosome) 1, 4, 7, 13 and 14. Supplementary Table S3 shows all European breeds with significantly negative *f*_3_ statistics. However, the influence and the main contributor of the introgression from Chinese pigs to European breeds varied between chromosomes. For example, none of the European breeds had been found to be introgressed on SSC1, while five breeds had significant introgression from Chinese pigs on SSC13 and 14. In addition, on SSC7 and 13, the most significantly negative *f*_3_ statistics were obtained when Chinese breeds such as Ganxi, Tongcheng and Shaziling, rather than Dongshan or Bamaxiang, were used as the putative source populations. Although Bamaxiang only provided the most significantly negative *f*_3_ statistic in Welsh on SSC13, it provided the second most significantly negative *f*_3_ values in several breeds and chromosomes.

### TreeMix analyses on simulated data

To investigate the effect of gene flow on the phylogeny structure of EUW and EUD, we conducted a TreeMix of data simulated for four scenarios. The first scenario allowed only migrations from EUD to EUW. Under this scenario, TreeMix could correctly infer population trees with EUD and EUW as two separate groups (Supplementary Fig. S9). Moreover, changing the extent of gene flows did not change the inferred tree structure. In the second scenario with migrations from EUW to EUD, we found that these migrations changed the position of EUW in the population tree (Supplementary Fig. S9). When the migration rate per generation was 2%, TreeMix inferred EUW and EUD as two separate groups; whereas, when the migration rate was increased to 4% and 6%, TreeMix placed EUW as a subgroup of the EUD. Thus, for this scenario, we further ran TreeMix allowing five migration events. As shown in Supplementary Fig. S10, TreeMix successfully corrected the tree structure at 4% and 6% of migration rate, by splitting EUW and EUD into two separate groups.

In the third and fourth scenarios, we further added migrations from ASD to EUD, in addition to gene flows between EUD and EUW. TreeMix analyses came to similar results as for the first two scenarios. The migrations from EUD to EUW led to two separate groups of EUD and EUW (Supplementary Fig. S11); and the migration from EUW to EUD led to the placement of EUW as a subgroup of EUD in all three cases of migration rate (Supplementary Fig. S11). In this scenario, we further ran TreeMix with 11 migration events. In all of these three cases, TreeMix correctly inferred the population structure with EUW and EUD separating as two groups (Supplementary Fig. S12).

## Discussion

### Introgression from Chinese pigs to European breeds

In this study, we confirmed the genetic contributions from Chinese pigs to European breeds. ADMIXTURE analyses indicated that most European breeds, including commercial and local breeds, were admixed because of introgression from Chinese pigs. The percentages of Chinese ancestry varied from 0.8% in Ibérico pigs to 23.2% in Large White when *K* = 2 (Fig. 2). The three-population tests identified four European breeds (White Duroc, Large White, British Saddleback and Landrace) with significant introgression from Chinese pigs. In addition, the TreeMix analysis (m = 8) presented migration edges from Chinese pigs to European breeds across all replicates, although the involved breeds and migration rates varied (Fig. 3 and Supplementary Fig. S6).

The percentages of Chinese ancestry were relatively high in most European breeds. In the ADMIXTURE analysis, European breeds, except for Ibérico and Mangalica, harboured about 20% of Chinese ancestry at *K* = 2. In the *F*_4_-ratio estimation, the percentages of Chinese ancestry reached about 25% in the four admixed breeds identified by the three-population tests. Our estimations confirm and refine the results of previous studies. The fractions of Chinese ancestry in European breeds in our ADMIXTURE analysis were similar to the results of^19^, who estimated the fractions at about 20% utilizing the same method based on whole-genome sequencing data. Our estimates are lower than an earlier finding^20^ based on *D*-statistics^21^ using Chinese Meishan pigs as a donor population, which suggested 38% of Chinese ancestry in Large White and Landrace. A previous mitochondrial study revealed that the average frequency of Asian mtDNA haplotypes across over European breeds was 29%, but varied considerably between breeds and lines with 12.8% in Landrace and 76.0% in Large White^6^. In addition, we noticed two traditional breeds, Ibérico and Mangalica, harboured little Chinese ancestry. This result is consistent with previous mitochondrial studies^6,7^, which showed that Ibérico pigs did not harbour Chinese mtDNA haplotypes. These results indicate that there has been little (if any) Chinese introgression into this breed. Thus, in the three-population tests and *F*_4_-ratio estimation, we used Ibérico pigs as the source population without introgression from Chinese pigs.

A previous mitochondrial study^6^ suggested that domestic pigs from South and East China were introgressed into European breeds as the Asian mtDNA haplotypes presented in European breeds were also found in South and East Chinese breeds. Recently, Ai *et al*. conducted an ADMIXTURE analysis using whole-genome sequencing data from 11 geographically diverse Chinese breeds and 5 European commercial breeds, as well as wild boars^12^. The result indicated that almost all of the introgressed Chinese haplotypes in European breeds came from South Chinese pigs, without any contribution from East Chinese breeds such as Meishan pigs. Given these two contradicting findings, studies using new and powerful methods are needed to refine the source of the introgression. In this study, we used both the TreeMix analysis and three-population test to infer the source of introgression. In the trees modelling eight migration events, we found that almost all gene flows to European pigs came from South Chinese breeds, including Dongshan and Bamaxiang. The exception to this was the observed gene flow from the common ancestor of Erhualian and Jinhua pigs to the common ancestor of Large Black, Berkshire and Gloucestershire Old Spots. In the three-population tests, we identified four European breeds with significantly negative *f*_3_ statistics. For these four breeds, the most significantly negative *f*_3_ statistics were all produced by using either Bamaxiang or Dongshan as one source population, as shown in Supplementary Table S2. These results supported that the South Chinese pigs played a main role in the introgression from Chinese pigs to European breeds. However, the contributions from Chinese pigs to European breeds were inconsistent across chromosomes. Among the five chromosomes with most markers, we observed that for some European breeds the most significantly negative *f*_3_ statistics were obtained by using Tongcheng and Shaziling from Central China and Ganxi from East China as one source population. Thus, we conclude that the introgression to European breeds mainly occurred from South Chinese pigs, while Chinese breeds from other regions also contributed to this process.

### Introgression from European pigs to Chinese breeds

For Chinese pigs, the ADMIXTURE analyses and three-population tests identified four breeds being admixed with European pigs, including Kele, Min, Sutai and Tibetan pigs. Except for Tibetan pigs, all these breeds have previously been reported to have introgression from European pigs^17^. As most European breeds are admixed with Chinese pigs, it is difficult to infer the source of the introgression to these four breeds. However, in the ADMIXTURE analysis with *K* = 6, Sutai pigs were found to have about 37% Chinese Meishan ancestry, 47% Duroc ancestry and a low proportion of other European breeds (Supplementary Fig. S13). This is in accordance with the report that the Sutai breed has been formed by crossing Chinese Taihu pigs (Meishan is one breed of Taihu pigs) and Duroc pigs^22^. As no European mtDNA haplotypes were detected in Min and Tibetan pigs in a previous study^6^, the introgression from European pigs to these two breeds is expected to be male-mediated.

### Admixture between European breeds

In the TreeMix analyses, among the ten replicates modelling eight migration events (Supplementary Fig. S6), eight of them detected a significant migration from the common ancestor of European *Sus scrofa* to Canarian pigs. This gene flow has been identified before, but in the opposite direction, which is impossible and might be caused by incorrect basal position inferred for the Canarian pigs in the tree modelled by TreeMix, as explained by the authors^23^. In addition, our result is consistent with historical records that Canarian pigs were crossed with international breeds, like Berkshire and Large Black, and autochthonous Spanish pigs^23^. Furthermore, we identified strong gene flows from Large White to White Duroc and from Duroc to White Duroc depending on the basal position of White Duroc (Fig. 3 and Supplementary Fig. S6). It indicated that White Duroc was produced by crossing Duroc with Large White, or other breeds closely related to Large White. ADMIXTURE analyses agreed well with this finding, by showing a large proportion of Duroc and Large White ancestries in White Duroc when *K* ≥ 13 (Supplementary Fig. S13).

### Gene flow between EUW and EUD

In the TreeMix analyses, EUW always located as a subgroup of EUD, even though we allowed eight migration events in the tree model. This pattern has also been shown in many previous studies^5,20,24^. It seems like that EUW are actually feral pigs, *i.e*., EUW are descended from domestic pigs while live in the wild. However, up to now, no other evidence supports that EUW descend from EUD. Another hypothesis could be that the admixture between EUW and EUD might influence the tree structure.

To explore this hypothesis, we conducted coalescence simulations under a simplified model. In the two scenarios with gene flows from EUW to EUD, TreeMix inferred EUW as a subgroup of EUD, except for the case with 2% of migration rate in the second scenario; in two other scenarios with gene flows in the opposite direction, TreeMix inferred EUW and EUD as two separate groups. Meanwhile, the introgression from EUW to EUD has been proven in our ADMIXTURE analysis at *K* = 3 (Fig. 2), as well as the full model of the history of Eurasian *Sus scrofa* inferred in a previous study^5^. Therefore, we suppose that the introgression from EUW to EUD might be the reason for the location of EUW among EUD. However, we were not able to determine the introgression came from which European wild boar population to which European domestic breed based on our genotyping data. As a result, we couldn’t further investigate the degree to which the phylogeny structure of EUD and EUW was affected by the introgression from EUW to EUD.

On the other hand, in ADMIXTURE analysis at *K* = 3, some EUW had up to 31% of EUD ancestry. At *K* = 2, these EUW also presented as high as 13% of Chinese ancestry. These results indicated that the gene flow from EUD to EUW led to the introgression of Chinese ancestry to EUW. This finding was consistent with previous studies^25,26^, where Chinese haplotypes had been found in EUW.

Domestication, breeding and admixture have shaped both phenotype and genomic variations presented within and between pig breeds. In this study, we clarify the relationship between pig breeds from China and Europe. We find that Chinese ancestry is common in both commercial and local breeds in Europe, and that the origin of this introgression is most likely the pig breeds in South China. However, East and Central Chinese pigs also contribute genomes to European breeds. Additionally, simulation studies prove that the introgression from EUW to EUD can result in the placement of EUW as a subgroup of EUD in phylogeny reconstruction. These results can facilitate genome-wide association studies and potentially genomic prediction by accounting for population structure, and the detection of selection signatures based on genomic admixture.

## Methods

### Date collection

We collected genotyping data from 1,135 samples in total, including 425 samples from the Dryad Digital Repository: http://dx.doi.org/10.5061/dryad.c2124 ^27,28^, 208 samples from the Dryad Digital Repository: http://dx.doi.org/10.5061/dryad.v6f1g ^29,30^, 304 samples from the Dryad Digital Repository: http://dx.doi.org/10.5061/dryad.53j31 ^17,31^, 61 samples kindly provided by^23^ and the last 137 samples were genotyped by Genoskan A/S (Tjele, Denmark). Genotyping was performed using the Porcine 60 K SNP panel. Supplementary Table S1 described the details of the dataset. The geographic distribution of Chinese pigs had been provided in a figure in Ai *et al*. paper^17^, except for Meishan pigs which originated from East China and was geographically close to Erhualian pigs. In the ADMIXTURE analysis at *K* = 2 (Fig. 2), Tibetan pigs both from Tibet and Gansu harboured low levels of European ancestry (on average 8% and 4%, respectively). Thus, we combined Tibetan pigs from Tibet and Gansu into one group to simplify the dataset. To merge these data, we retained markers that were common to all datasets, and excluded markers fixed in the whole dataset. After deleting SNPs without a known genomic location in the *Sus scrofa* build 10.2^20^, 30,549 autosomal SNPs remained with an average adjacent marker spacing of 64.7 ± 79.8 kb. Thirteen samples were excluded because of low genotype call rates (<95%). In addition, we collected whole-genome sequencing data from two Sumatran wild boars^32^, and extracted genotypes of all the SNPs which were in the merged dataset. We used these two Sumatran wild boars as an outgroup in the TreeMix analyses and *F*_4_-ratio estimation.

### PCA analysis

PCA was conducted on all EUW using SMARTPCA, part of EIGENSOFT 6.0.1^33^. The analysis clustered wild boars into several groups. According to this result, we conducted the relatedness test and following analyses on each European wild boar group.

### Relatedness test

To ensure independence among the collected individuals, relatedness tests were conducted in PLINK^34^. For EUW, we performed the test for each group according to the groupings determined by PCA; for Chinese wild boars, we performed the test by treating all individuals as one group, because all of them were sampled from Jiangxi Province, China^17^; for domestic pigs, we performed the test separately for each breed. To reduce the dependency of SNPs, in each test, we excluded SNPs with MAF <0.05 and generated a pruned dataset with SNPs in approximate linkage equilibrium (–indep-pairwise 50 5 0.5, *i.e*., remove one of a pair of SNPs with a pairwise r^2^ > 0.5 within a window of 50 SNPs, and shift the window by a step of 5 SNPs). A pair of individuals with an identity by decent value (pi-hat) >0.3 was considered to be closely related, and one of them was removed from the analyses. Supplementary Table S1 shows the number of individuals removed due to close relatedness.

### ADMIXTURE analysis

ADMIXTURE 1.23^16^ was used to evaluate ancestry proportions for *K* ancestral populations. We ran ADMIXTURE with 5-fold cross-validation for the values of *K* from 1 to 40 to examine the patterns of ancestry and admixture in our dataset. The lowest 5-fold cross-validation error was used as the indicator of the most probable *K*-value, as suggested by the authors of ADMIXTURE.

### TreeMix analysis

TreeMix software first builds a maximum likelihood tree of sampled populations by using a Gaussian approximation of genetic drift of allele frequencies. Subsequently, it identifies populations whose genetic covariance is underestimated by the model. Migration events are added to improve fitness of the model. TreeMix 1.12^18^ was used to infer the relationships (splitting and mixing) between Eurasian *Sus scrofa* populations by using Sumatran wild boars as an outgroup. Because the proportion of introgression from Chinese pigs has been reported to vary between lines within the same European breed^6^, we separated swine breeds with at least two data resources into groups corresponding to the resources. For example, Large White was separated into three groups, including LW_A, LW_W and LW_G, to indicate individuals from Ai *et al*.^17^, Wilkinson *et al*.^28^ and Goedbloed *et al*.^30^. To simplify the tree model, we further removed the four admixed Chinese breeds Kele, Sutai, Min, and Tibetan pigs. The block size was set to 200 SNPs to ensure independency between blocks. Between 0 and 10 migration events were modelled. To verify the consistency of migration edges, ten independent replicates were run for the model with eight migration events. Phylogenetic networks for tree models were visualized using the R package ggtree^35^.

### Three-population test

The three-population test^36^ was used to detect population admixture, and implemented in the *threepop* program of TreeMix^18^. This test can verify if the target population X is related to the populations A and B through a simple tree or an admixture of A and B. For a simple bifurcating tree, the product of the frequency differences (*f*_3_(X; A, B) statistic) between A and X, and B and X, is expected to be positive. The product can be negative only if the population X has ancestry from populations related to both A and B. Thus, a significantly negative value of the *f*_3_ statistic provides evidence of admixture in the history of the population X. To assess the statistical significance of the three-population test, we used a Block Jackknife^37^ with a window of 200 SNPs to obtain the standard error of the test, which was then used to generate a *Z* score. The window size was chosen to span much larger than the typical extent of linkage disequilibrium. A large negative *Z* score (*Z* < −2) indicated a significant evidence of admixture.

This test was conducted for each Chinese and European breed under the scenarios *f*_3_ (ASD1; ASD2 or ASW, IB or EUW) and *f*_3_ (EUD; IB or EUW, ASD). Here, ASD was an Asian domestic breed, ASW was an Asian wild boar group, EUD was a European domestic breed, EUW was a European wild boar group and IB was Ibérico pigs. For EUW, we only included individuals with less than 5% of Chinese ancestry in the ADMIXTURE analysis at *K* = 2.

### *F*_4_-ratio estimation

The *f*_4_ (A, B; C, D) statistic is the product of the frequency differences between the populations A and B, and populations C and D. The *F*_4_-ratio estimation^36^, calculated as the ratio of two *f*_4_ statistics, was used to estimate the mixture proportions in an admixed population. In our case, to estimate the proportion of Chinese ancestry in a European domestic pig breed, we used two Sumatran wild boars as an outgroup and computed the ratio of two *f*_4_ statistics:

1 $${f}_{4}-ratio=\frac{{f}_{4}(EUW,Sum;EUD,BMX)}{{f}_{4}(EUW,Sum;IB,BMX)}=\alpha $$

where EUD was a European domestic breed; EUW was a European wild boar population; Sum was two Sumatran wild boars; IB was Ibérico pigs to represent a European domestic breed without admixture with Chinese pigs; BMX was Bamaxiang pigs to represent the origin of Chinese introgression; α was the fraction of IB-like ancestry in the population EUD. The fraction of Chinese ancestry was then calculated as 1-α. For EUW, we only included individuals with less than 5% of Chinese ancestry in the ADMIXTURE analysis at *K* = 2. The standard errors were computed using the Block Jackknife^37^ with a window size of 200 SNPs.

### Simulations

**Figure 4:**
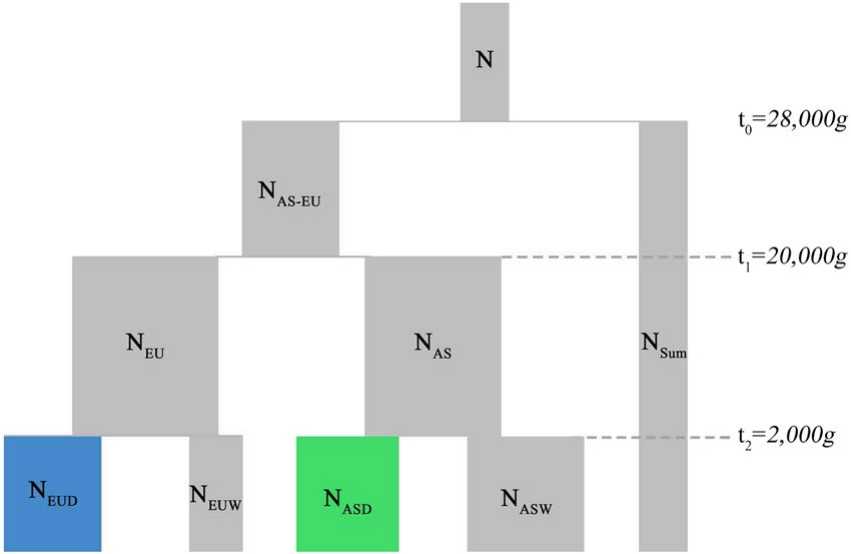
Illustration of the simulated model. Effective population sizes: *N* = *N*_*Sum*_ = 10,000, *N*_*EU*_ = 175,000, *N*_*AS*_ = 170,000, *N*_*EUD*_ = 20,000, *N*_*EUW*_ = 8,000, *N*_*ASD*_ = 26,000 and *N*_*ASW*_ = 36,000.

To model the history of Eurasian *Sus scrofa* (Fig. 4), we performed coalescent simulations using MaCS^38^. Parameters of *N*_*EU*_, *N*_*EUW*_, *N*_*EUD*_, *N*_*AS*_, *N*_*ASW*_ and *N*_*ASD*_ were selected from the full model of Eurasian wild boars and domestic pigs inferred by an approximate Bayesian computation method^5^. We assumed one generation every five years, and a mutation rate *μ* = 1 × 10^−8^/*bp* and a recombination rate *r* = 1 × 10^−8^/*bp*. To reflect the formation of domestic breeds and wild boar groups, we produced eight European domestic breeds by population isolations from EUD, three Asian breeds by population isolations from ASD, seven European wild boar groups by population isolations from EUW and one Asian wild boar group by population isolation from ASW. These population isolations happened 40–60 generations BP (before present). Breed formation and subsequent artificial selection resulted in a sharp decrease of effective population size. Thus, we set the effective population sizes of isolated populations to 2,000. Under this model, we studied four scenarios involving different patterns of continuous introgression, including gene flow (I) from EUD to EUW, (II) from EUW to EUD, (III) from EUD to EUW and from ASD to EUD, (IV) from EUW to EUD and from ASD to EUD. The gene flow from ASD to EUD started about 40 generations BP, where six European breeds received migrants from one Asian breed. The migration rate per generation varied from 1% to 2% in each European breed and continued for 10 generations. The gene flow from EUW to EUD started about 30 generations BP, where five European domestic breeds received migrants from five different European wild boar groups at the same time. For the migration rate, we tested three different fractions (2%, 4% and 6% each generation) and continued for 10 generations. The gene flow from EUD to EUW was the same as from EUW to EUD, except in the opposite direction. Thus, in each scenario, we had three cases with different fractions of migration between EUD and EUW. In each case, we simulated 1,000 regions of 50 kb each and generated 40 chromosomes from each population. To run TreeMix analyses on the simulated data, we used Sumatran wild boars as an outgroup, and used a block size of 5,000 SNPs.

### Data accessibility

Genotyping data are from 425 samples from the Dryad Digital Repository (http://dx.doi.org/10.5061/dryad.c2124), 208 samples from the Dryad Digital Repository (http://dx.doi.org/10.5061/dryad.v6f1g), 304 samples from the Dryad Digital Repository (http://dx.doi.org/10.5061/dryad.53j31), 61 samples from the study: “A high throughput genotyping approach reveals distinctive autosomal genetic signatures for European and Near Eastern wild boar” (data available on reasonable request), and the last 137 samples will be available from the Dryad Digital Repository after the paper is accepted for publication.

## Electronic supplementary material

Supplementary Information
