# Supplementary material for: Population admixture in Chinese and European Sus scrofa
Source: Sci Rep. 2017 Oct 13;7:13178. doi: 10.1038/s41598-017-13127-3 (PMC5640611; doi:10.1038/s41598-017-13127-3)
Supplement: Supplementary file 1 — Supplementary Information [file 41598_2017_13127_MOESM1_ESM.pdf]

Population admixture in Chinese and European *Sus scrofa*

Minhui Chen[1, 2], Guosheng Su[1*], Jinluan Fu[2], Qin Zhang[2], Aiguo Wang[2], Mogens Sandø Lund[1], Bernt Guldbrandtsen[1]

[1] Center for Quantitative Genetics and Genomics, Department of Molecular Biology and Genetics, Aarhus University, Tjele, Denmark

[2] Department of Animal Genetics, Breeding and Reproduction, China Agricultural University, Beijing, China

[*] Corresponding author

E-mail: guosheng.su@mbg.au.dk

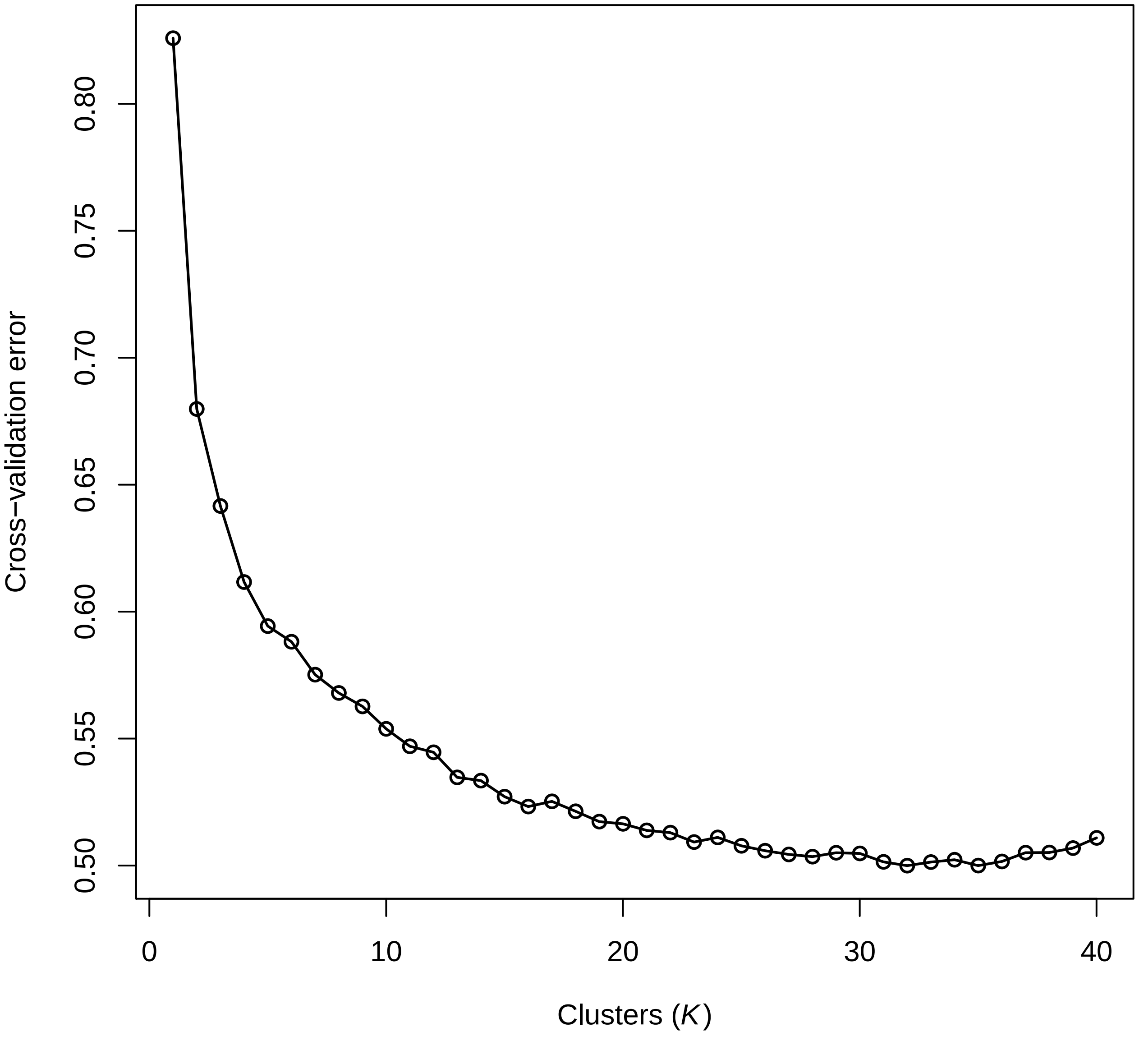

Fig S1. Cross-validation errors for various *K* values in the ADMIXTURE analyses. The *K* value with the lowest cross-validation error indicates the optimal number of ancestral populations.

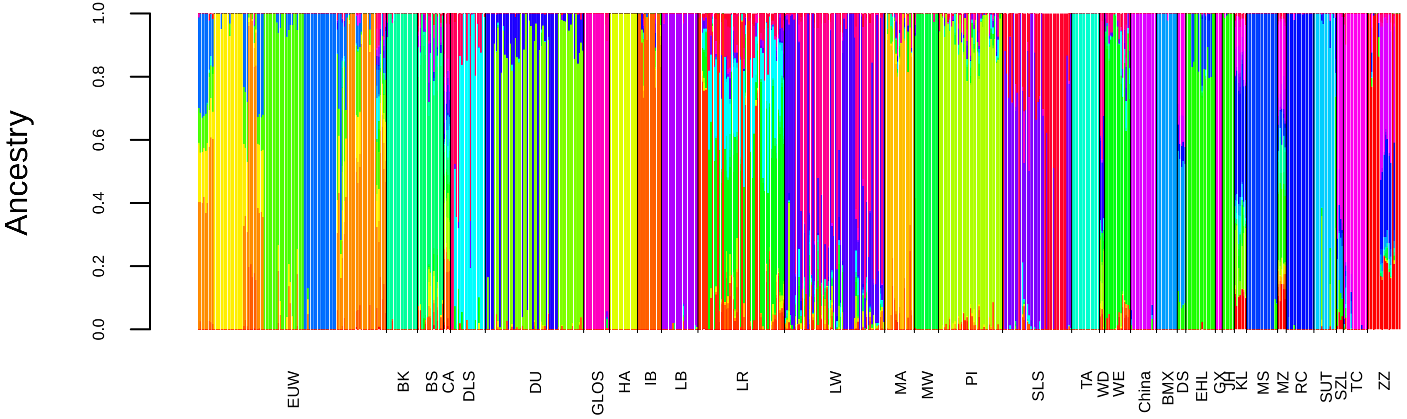

Fig S2. Ancestry composition using 32 ancestral populations (*K* = 32) in ADMIXTURE analysis.

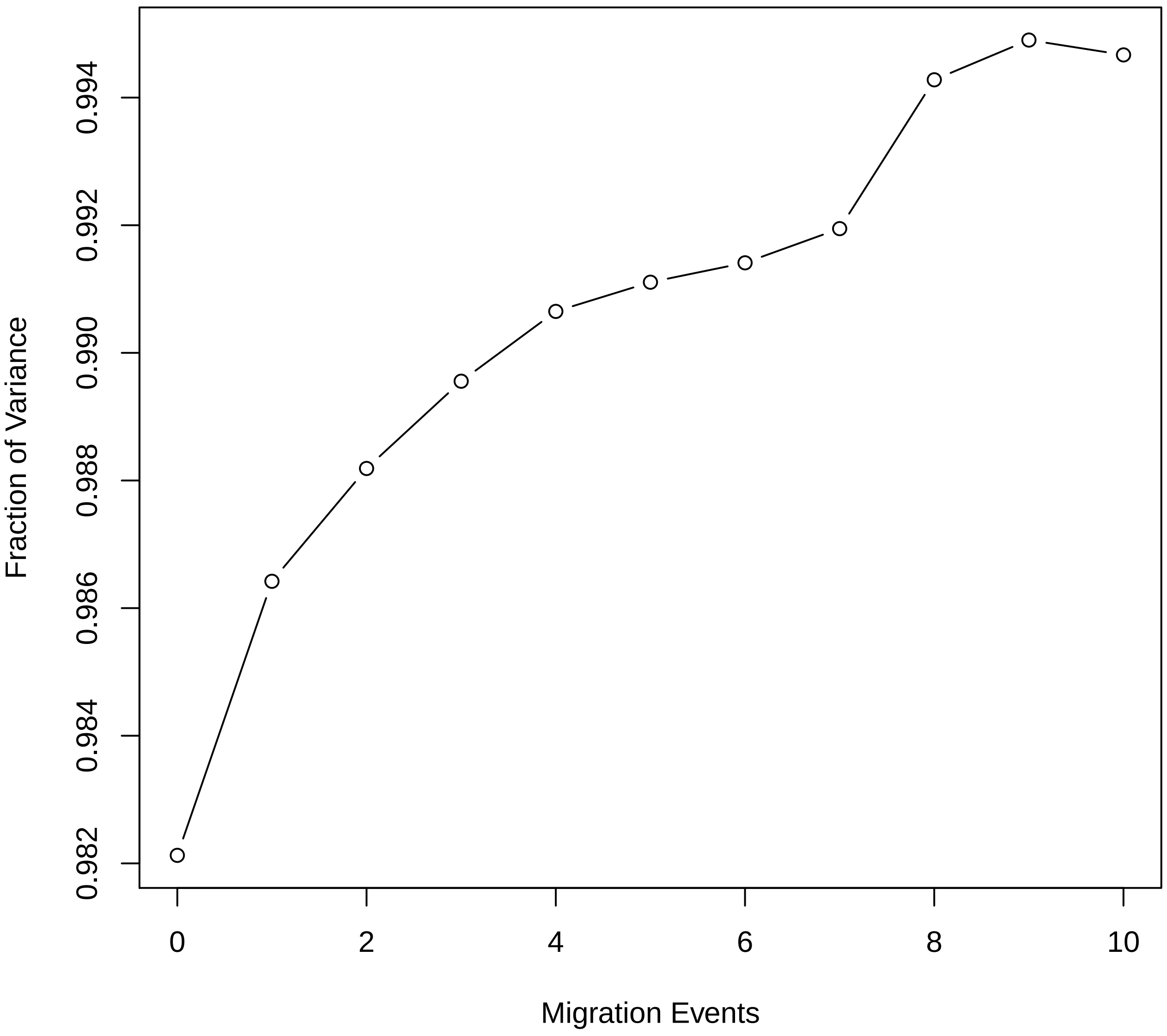

Fig S3. The fractions of variance in relatedness between populations explained by phylogenetic models with 0 to 10 migration events.

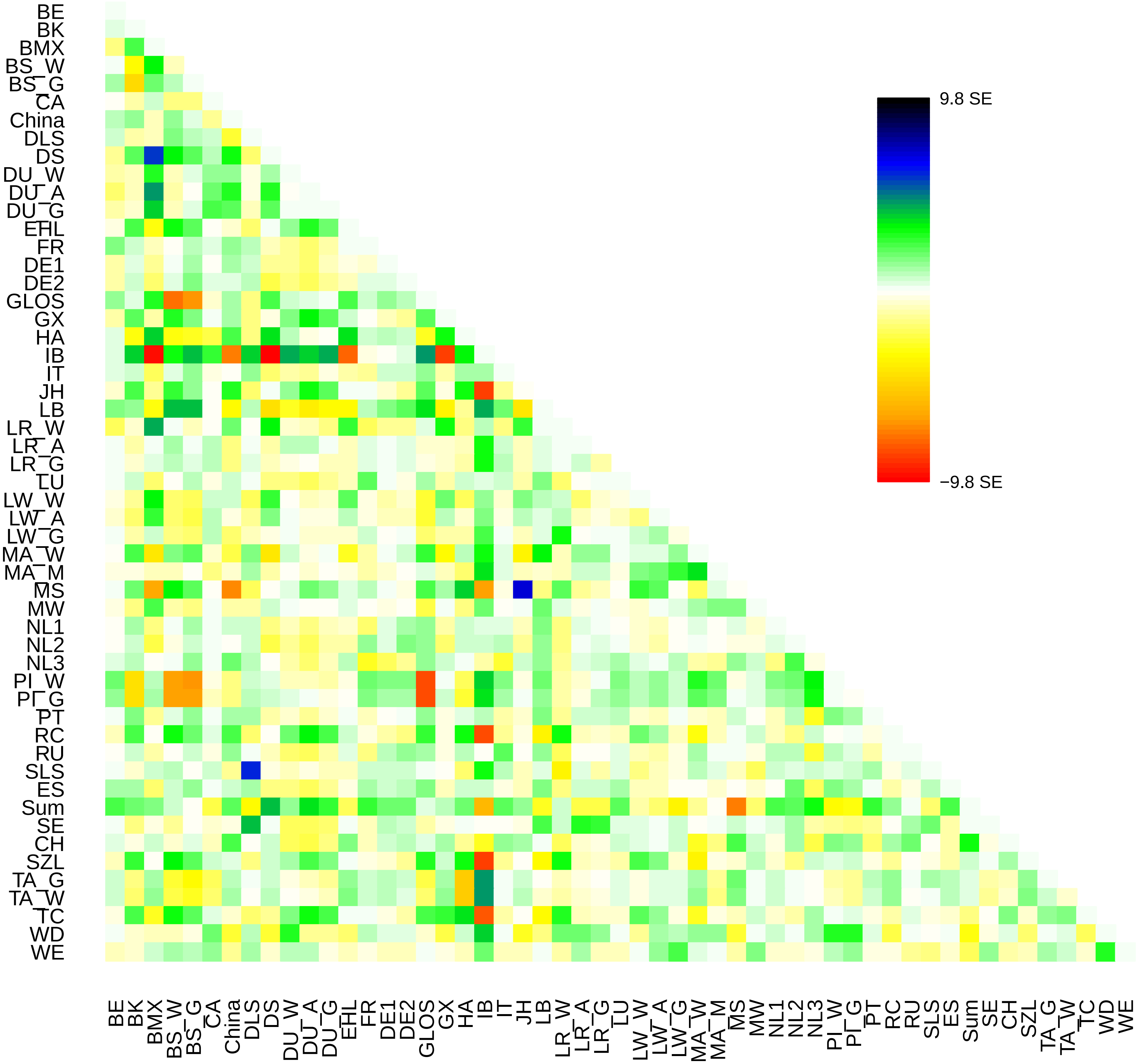

Fig S4. Residuals fit from the phylogenetic network with 8 migration events. Each cell indicates the residual covariance between each pair of population scaled by the average standard error across all pairs. Residuals above zero represent populations that are closer in the data than in the model, and thus are candidates for migration.

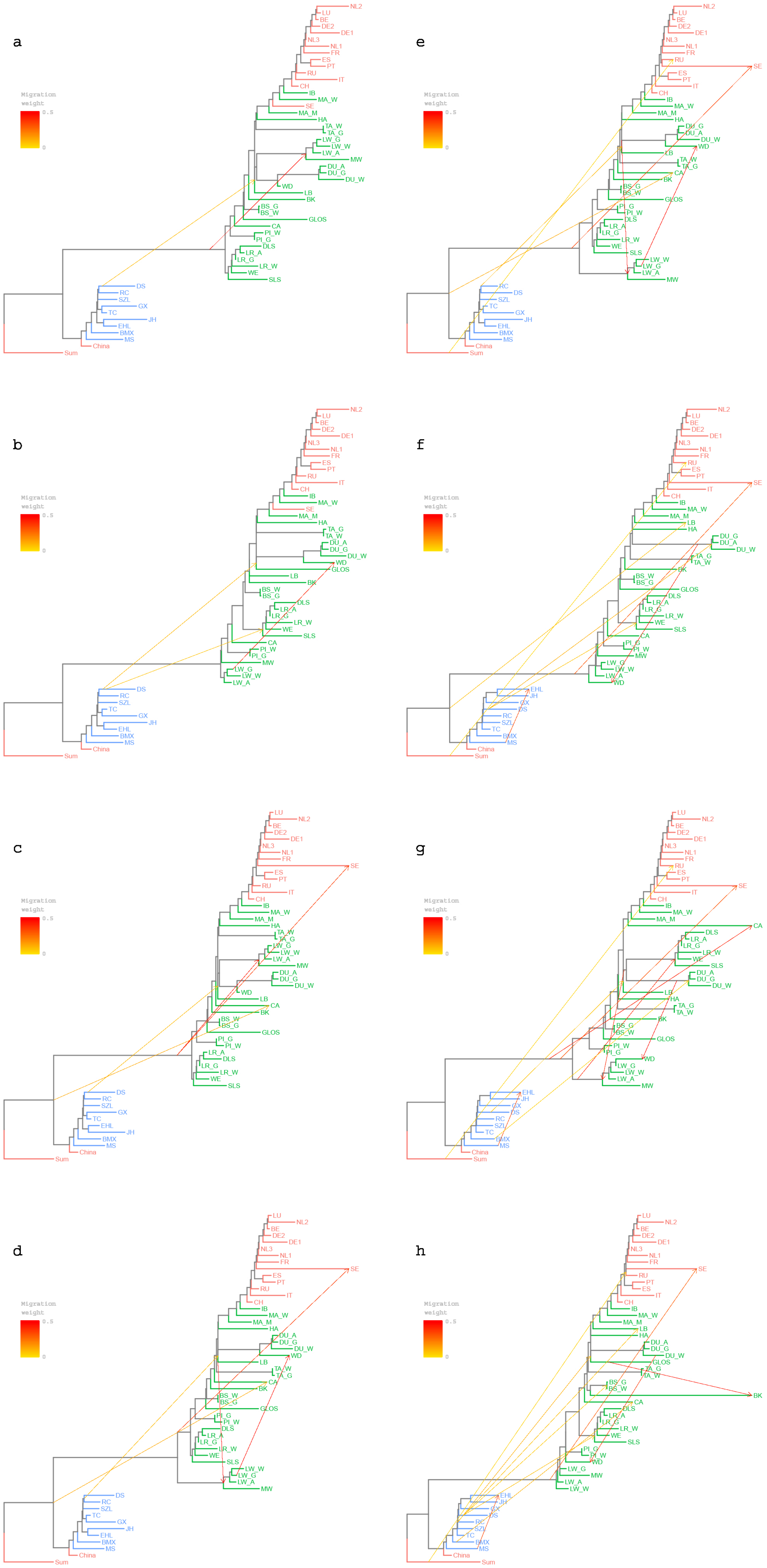

Fig S5. Phylogenetic networks with 2 to 7, 9 and 10 migration events. Wild boar populations were coloured as red; Chinese domestic breeds were coloured as blue; and European domestic breeds were coloured as green. Migration edges were coloured according to percent of ancestry received from the donor population. Populations of the same breed were divided into groups corresponding to the resources of data. European wild boars were separated into groups according to the result of principal component analysis. Sum represented Sumatran wild boars. BE: Belgium; CH: Switzerland; (DE1, DE2): Germany; ES: Spain; FR: France; IT: Italy; LU: Luxemburg; (NL1, NL2 and NL3): Netherlands; PT: Portugal; RU: Russia; SE: Sweden.

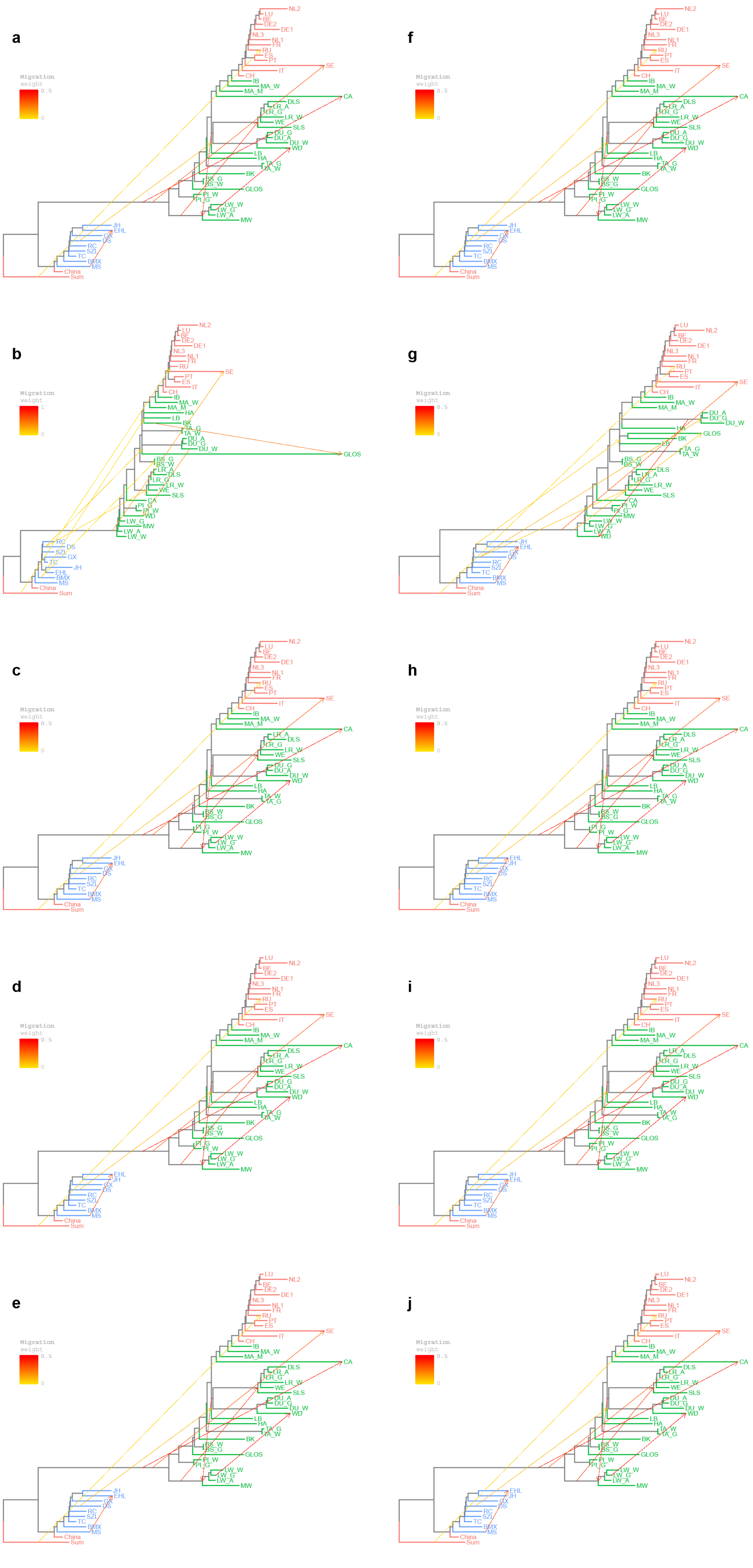

Fig S6. Ten independent replicated phylogenetic networks for tree models with eight migration events. Wild boar populations were coloured as red; Chinese domestic breeds were coloured as blue; and European domestic breeds were coloured as green. Migration edges were coloured according to percent of ancestry received from the donor population. Populations of the same breed were divided into groups corresponding to the resources of data. European wild boars were separated into groups according to the result of principal component analysis. Sum represented Sumatran wild boars. BE: Belgium; CH: Switzerland; (DE1, DE2): Germany; ES: Spain; FR: France; IT: Italy; LU: Luxemburg; (NL1, NL2 and NL3): Netherlands; PT: Portugal; RU: Russia; SE: Sweden.

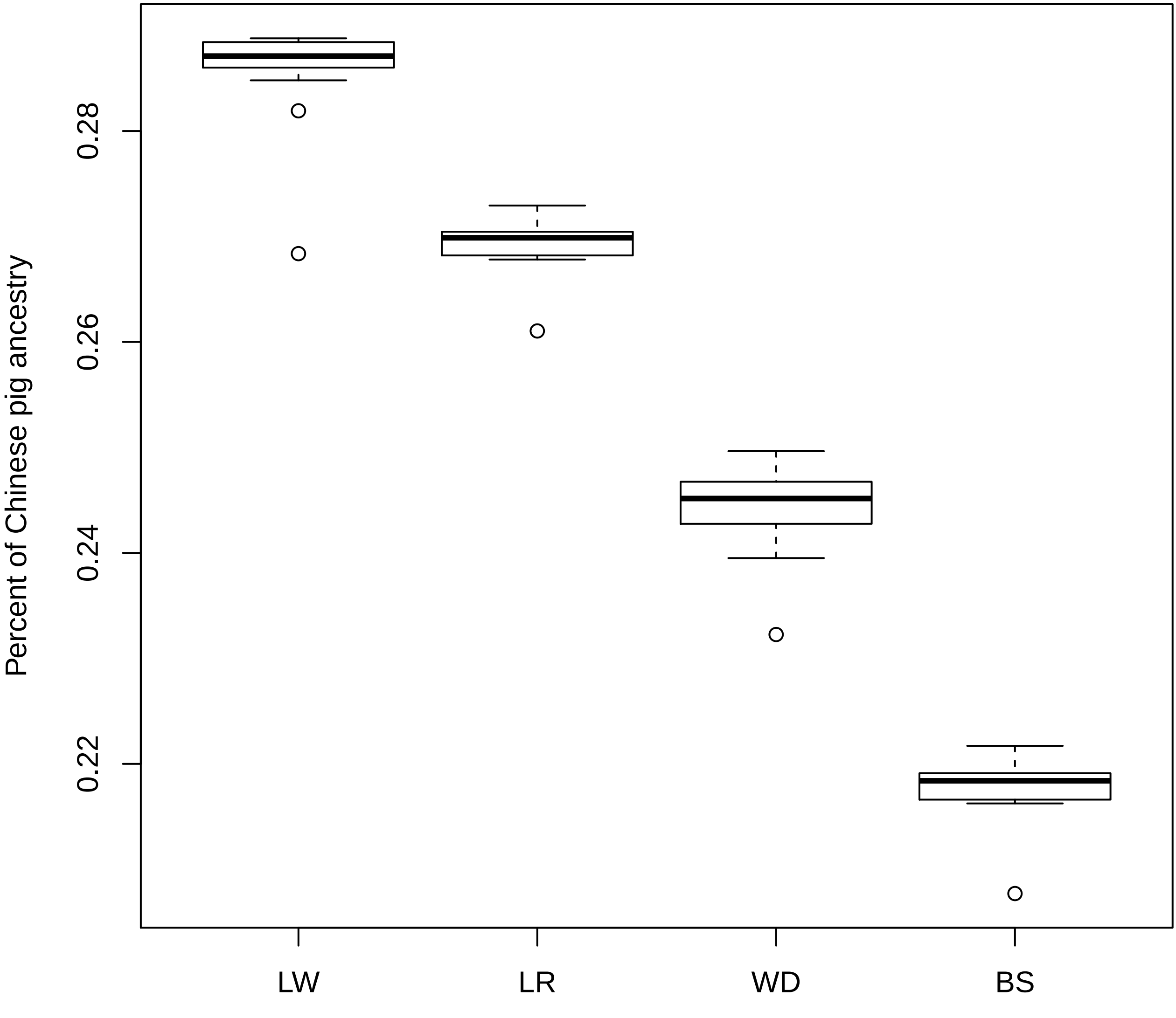

Fig S7. *F*4-ratio estimations of Chinese pig ancestry in Large White (LW), Landrace (LR), White Duroc (WD) and British Saddleback (BS) by using various European wild boar populations as one source population.

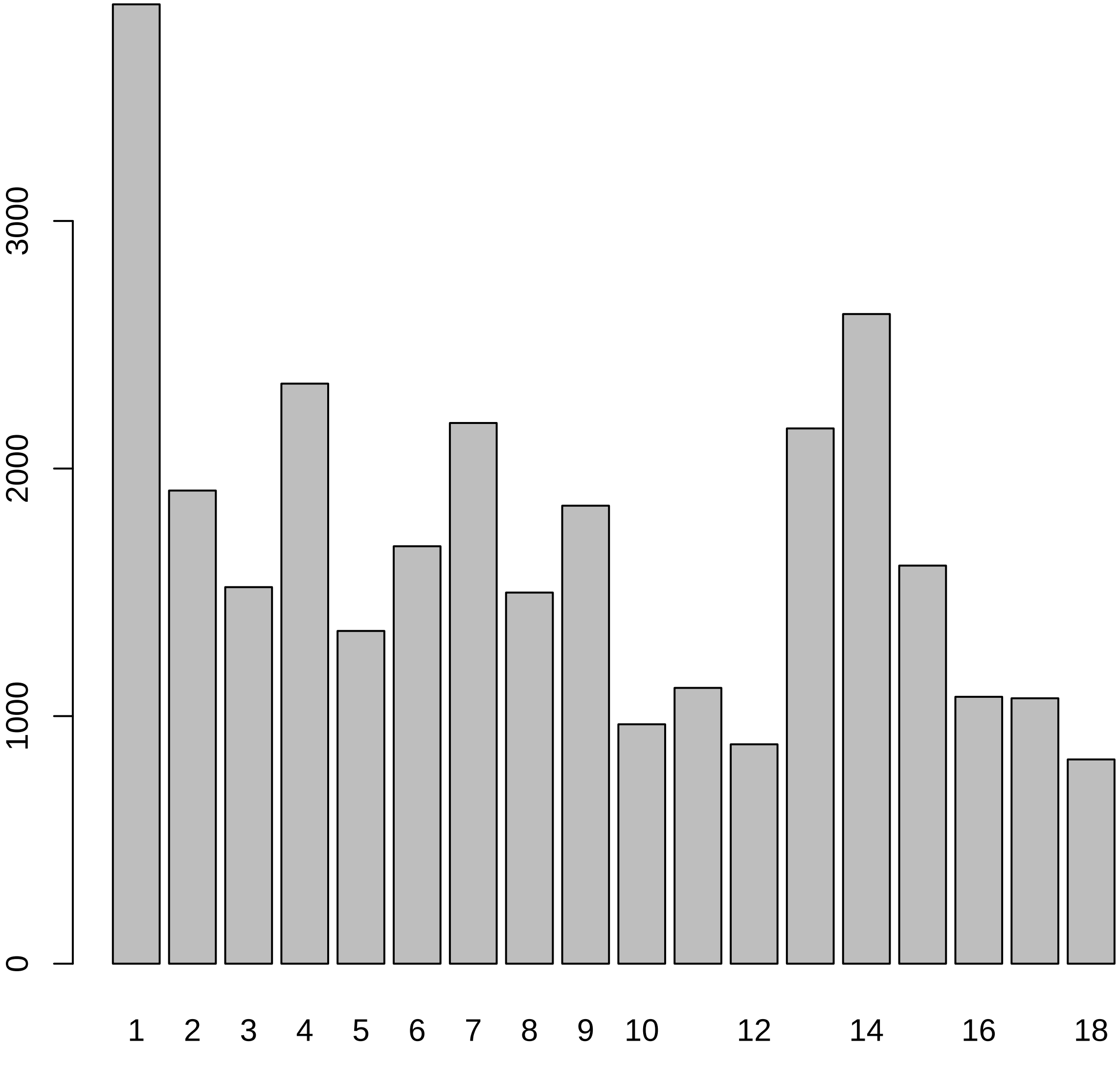

Fig S8. The distribution of SNP numbers across chromosomes.

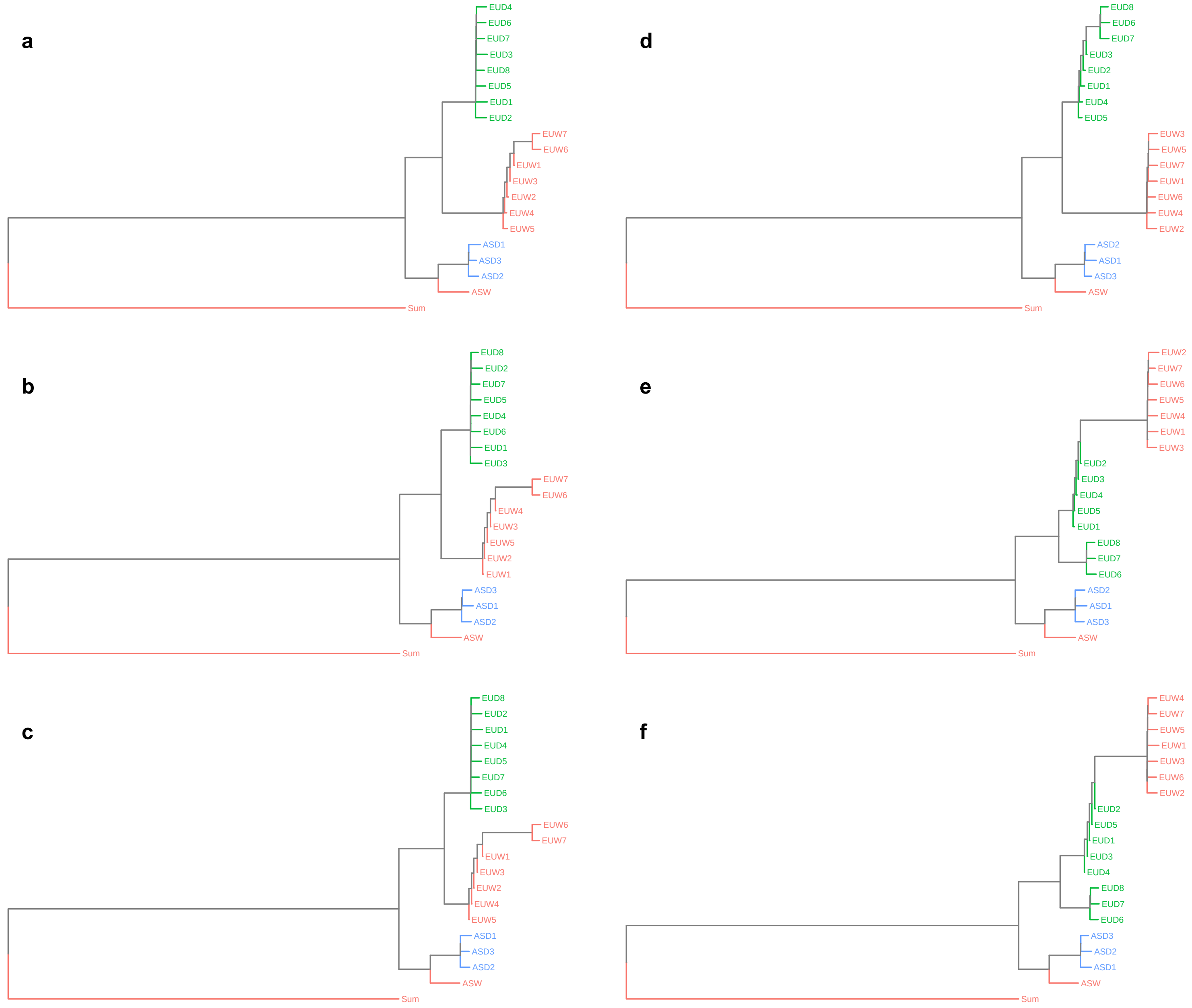

Fig S9. Tree models for simulations with gene flows between EUW and EUD. (a-c) Simulations with gene flows from EUD to EUW, with migration rate of 2%, 4% and 6%. (d-f) Simulations with gene flows from EUW to EUD, with migration rate of 2%, 4% and 6%.

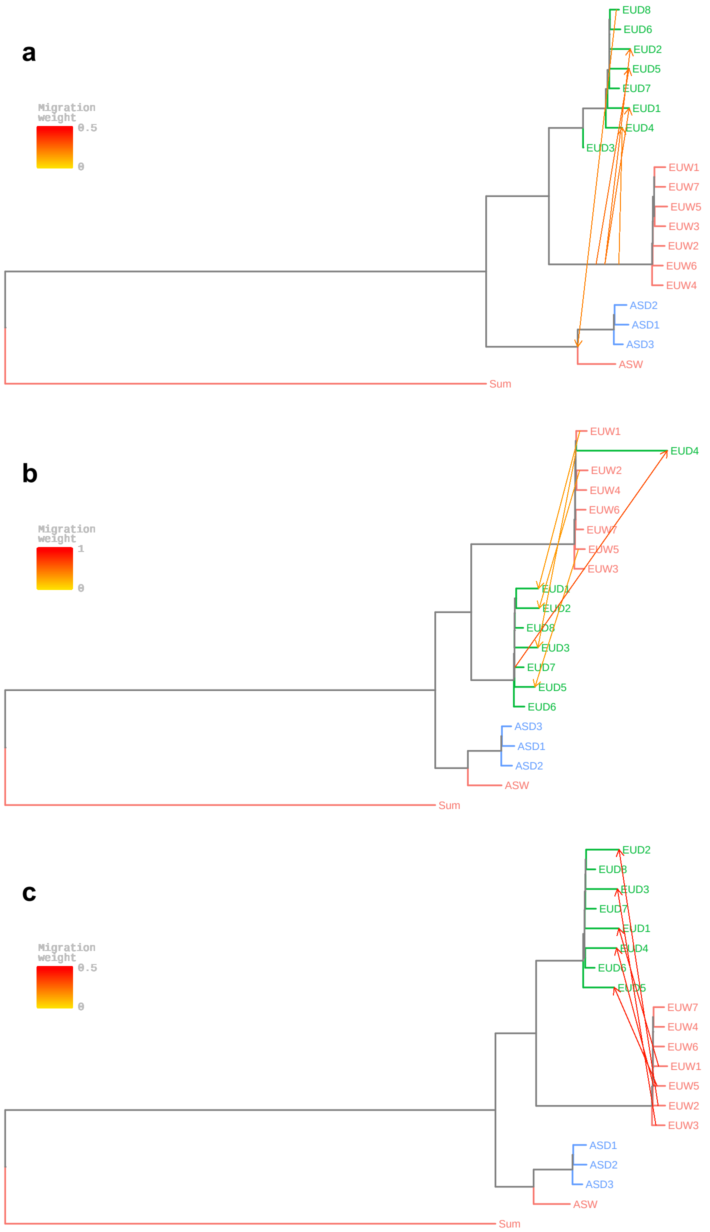

Fig S10. Tree models with five migration events for simulations with gene flows from EUW and EUD. (a-c) Simulations with gene flows from EUW to EUD, with migration rate of 2%, 4% and 6%.

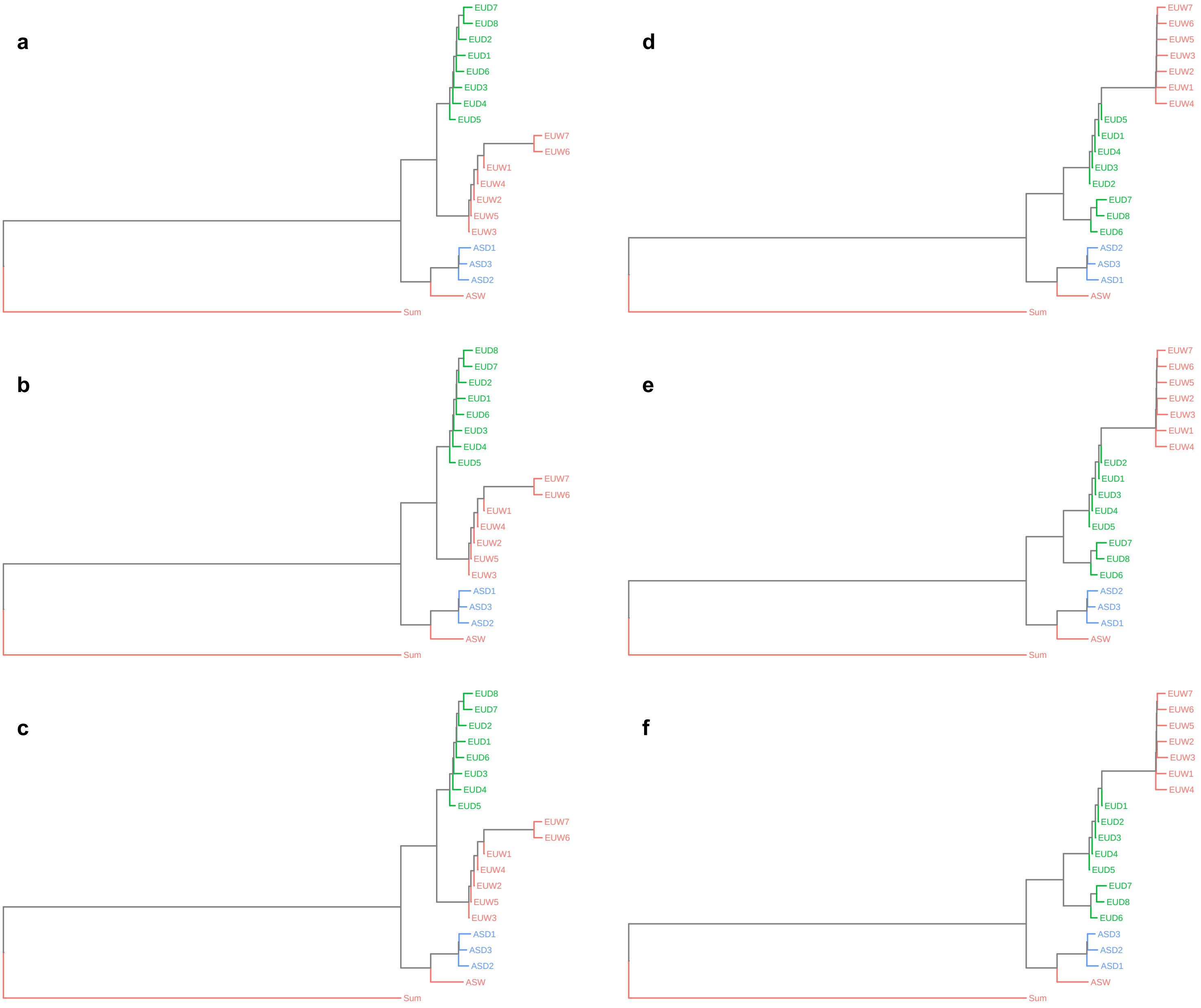

Fig S11. Tree models for simulations with gene flows between EUW and EUD and introgression from ASD to EUD. (a-c) Simulations with gene flows from EUD to EUW, with migration rate of 2%, 4% and 6%. (d-f) Simulations with gene flows from EUW to EUD, with migration rate of 2%, 4% and 6%.

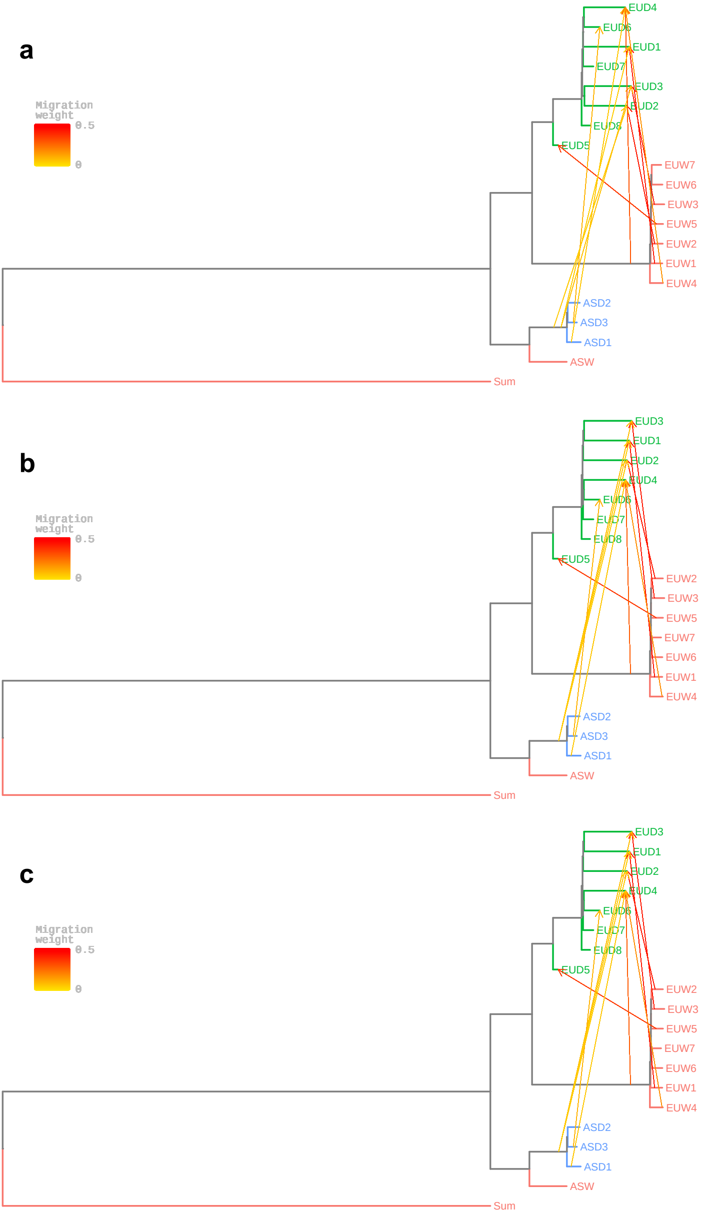

Fig S12. Tree models with 11 migration events for simulations with gene flows from EUW and EUD and from ASD to EUD. (a-c) Simulations with gene flows from EUW to EUD, with migration rate of 2%, 4% and 6%.

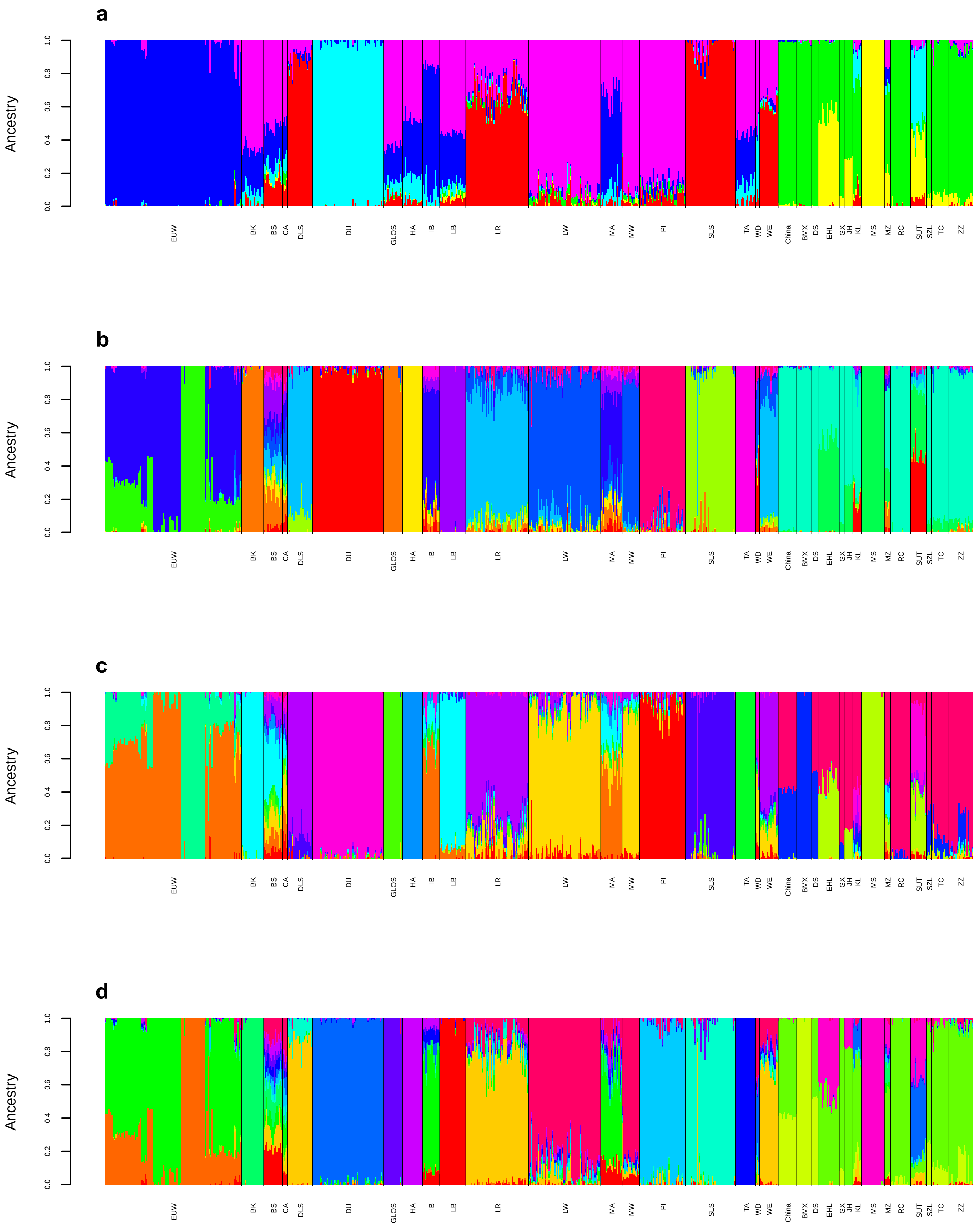

Fig S13. Ancestry models with 6, 13, 14 and 15 ancestral populations (*K* = 6, 13, 14 and 15).

Supplementary Table S1. Overview of analysed populations and sample sizes.

Domestic / Wild pigs		Pre QC[1]	Genotype call rate < 95%	pi-hat > 0.3	After QC[2]	Ref.
**European domestic pigs**						
Berkshire	BK	29	0	11	18	Wilkinson et al., 2013
British Saddleback	BS	50	1	34	15	Wilkinson et al., 2013; Goedbloed et al., 2013
Canarian	CA	4	0	0	4	Manunza et al., 2013
Danish Landrace	DLS	37	1	16	20	
Duroc[3]	DU	66	0	9	57	Wilkinson et al., 2013; Goedbloed et al., 2013; Ai et al., 2013
Gloucestershire Old Spots	GLOS	24	0	9	15	Wilkinson et al., 2013
Hampshire[3]	HA	30	0	14	16	Wilkinson et al., 2013
Ibérico	IB	16	0	2	14	Manunza et al., 2013
Large Black	LB	30	0	9	21	Wilkinson et al., 2013
Landrace	LR	67	0	17	50	Wilkinson et al., 2013; Goedbloed et al., 2013; Ai et al., 2013
Large White	LW	71	0	13	58	Wilkinson et al., 2013; Goedbloed et al., 2013; Ai et al., 2013
Mangalica	MA	46	0	29	17	Wilkinson et al., 2013; Manunza et al., 2013
Middle White	MW	30	3	13	14	Wilkinson et al., 2013
Pietrain	PI	46	0	9	37	Wilkinson et al., 2013; Goedbloed et al., 2013
Pied Landrace	SLS	100	0	60	40	
Tamworth	TA	50	0	34	16	Wilkinson et al., 2013; Goedbloed et al., 2013
White Duroc	WD	5	0	2	3	Ai et al., 2013
Welsh	WE	33	0	18	15	Wilkinson et al., 2013
**Chinese domestic pigs**						
Bamaxiang	BMX	16	0	4	12	Ai et al., 2013
Dongshan	DS	15	0	10	5	Ai et al., 2013
Erhualian	EHL	32	0	15	17	Ai et al., 2013
Ganxi	GX	13	0	9	4	Ai et al., 2013
Jinhua	JH	13	0	6	7	Ai et al., 2013

Kele	KL	10	0	3	7	Ai et al., 2013
Meishan	MS	24	0	6	18	Wilkinson et al., 2013
Min	MZ	15	0	10	5	Ai et al., 2013
Rongchang	RC	18	0	2	16	Ai et al., 2013
Sutai	SUT	15	1	1	13	Ai et al., 2013
Shaziling	SZL	11	0	7	4	Ai et al., 2013
Tongcheng	TC	16	0	2	14	Ai et al., 2013
Tibetan	ZZ	50	0	31	19	Ai et al., 2013
**Wild boars**						
Belgium	BE	6	0	0	6	Manunza et al., 2013
France	FR	6	0	3	3	Wilkinson et al., 2013
Germany	DE	32	6	6	20	Goedbloed et al., 2013
Italy	IT	5	0	0	5	Wilkinson et al., 2013
Luxemburg	LU	4	0	0	4	Goedbloed et al., 2013
Netherlands	NL	58	1	9	48	Wilkinson et al., 2013; Goedbloed et al., 2013
Portugal	PT	6	0	1	5	Wilkinson et al., 2013
Russia	RU	4	0	1	3	Manunza et al., 2013
Spain	ES	11	0	2	9	Manunza et al., 2013
Sweden	SE	2	0	0	2	Wilkinson et al., 2013
Switzerland	CH	4	0	0	4	Wilkinson et al., 2013
China		15	0	0	15	Ai et al., 2013

[1] Sample sizes before quality control

[2] Samples sizes after quality control

[3] For convenience, Duroc and Hampshire were indicated as European breeds, although they originated in America.

Supplementary Table S2. Three-population tests for Chinese and European domestic breeds.

Target Population	Mixing populations	f_3	*Z*-score
Large White	Ibérico, Bamaxiang	-0.014	-6.6
British Saddleback	Ibérico, Dongshan	-0.012	-5.22
Landrace	Ibérico, Dongshan	-0.011	-5.02
White Duroc	Ibérico, Bamaxiang	-0.012	-4.74
Kele	Ibérico, Rongchang	-0.044	-30.21
Sutai	Ibérico, Erhualian	-0.028	-14.63
Min	Ibérico, Jinhua	-0.015	-7.11
Tibetan	Ibérico, Rongchang	-0.005	-5.61

[*]The presented are all populations with at least one significantly negative f3 statistic (Z-score < -2), the names of the putative mixing populations that give rise to the most significantly negative f3 statistic, the value of f3 statistic, and the Z-score. The populations labelled as “mixing” populations are those that give the most significantly negative f3 statistic, and are not necessarily the populations that actually mixed historically.

Supplementary Table S3. Three-population tests on SSC1, 4, 7, 13 and 14 for European domestic breeds.

SSC	Target Population	Mixing popualtions	f_3	*Z*-score
4	Landrace	Ibérico, Dongshan	-0.012	-2.01
7	Large White	Ibérico, Ganxi	-0.015	-2.53
13	White Duroc	Ibérico, Shaziling	-0.017	-3.64
13	British Saddleback	Ibérico, Tongcheng	-0.019	-3.2
13	Large White	Ibérico, Dongshan	-0.021	-2.45
13	Landrace	Ibérico, Ganxi	-0.016	-2.44
13	Welsh	Ibérico, Bamaxiang	-0.014	-2.23
14	Large White	Ibérico, Dongshan	-0.032	-4.26
14	British Saddleback	Ibérico, Dongshan	-0.032	-4.08
14	White Duroc	Ibérico, Dongshan	-0.027	-3.35
14	Mangalica	Ibérico, Dongshan	-0.022	-3.24
14	Canarian	Ibérico, Dongshan	-0.022	-2.55

[*]The listed are populations with at least one significantly negative f3 statistic (Z-score < -2) for each chromosome, the names of the putative mixing populations that give rise to the most significantly negative f3 statistic, the value of f3 statistic, and the Z-score. The populations labelled as “mixing” populations are those that give the most significantly negative f3 statistic, and are not necessarily the populations that actually mixed historically.
